# Structural basis of the substrate preference towards CMP for a thymidylate synthase MilA involved in mildiomycin biosynthesis

**DOI:** 10.1038/srep39675

**Published:** 2016-12-21

**Authors:** Gong Zhao, Cheng Chen, Wei Xiong, Tuling Gao, Zixin Deng, Geng Wu, Xinyi He

**Affiliations:** 1 State Key Laboratory of Microbial Metabolism and School of Life Sciences and Biotechnology, Shanghai Jiao Tong University, Shanghai 200030 China

## Abstract

Modified pyrimidine monophosphates such as methyl dCMP (mdCMP), hydroxymethyl dUMP (hmdUMP) and hmdCMP in some phages are synthesized by a large group of enzymes termed as thymidylate synthases (TS). Thymidylate is a nucleotide required for DNA synthesis and thus TS is an important drug target. In the biosynthetic pathway of the nucleoside fungicide mildiomycin isolated from *Streptomyces rimofaciens ZJU5119*, a cytidylate (CMP) hydroxymethylase, MilA, catalyzes the conversion of CMP into 5′-hydroxymethyl CMP (hmCMP) with an efficiency (*k*_cat_/*K*_M_) of 5-fold faster than for deoxycytidylate (dCMP). MilA is thus the first enzyme of the TS superfamily preferring CMP to dCMP. Here, we determined the crystal structures of MilA and its complexes with various substrates including CMP, dCMP and hmCMP. Comparing these structures to those of dCMP hydroxymethylase (CH) from T4 phage and TS from *Escherichia coli* revealed that two residues in the active site of CH and TS, a serine and an arginine, are respectively replaced by an alanine and a lysine, Ala176 and Lys133, in MilA. Mutation of A176S/K133R of MilA resulted in a reversal of substrate preference from CMP to dCMP. This is the first study reporting the evolution of the conserved TS in substrate selection from DNA metabolism to secondary nucleoside biosynthesis.

5-Hydroxymethyl cytosine (5hmC), also known as the ‘sixth base’, was discovered in mammalian and T-even phage DNA^1^,^2^. 5hmC in mammalian DNA is produced post-replicatively by the Tet-catalyzed oxidation of 5-methyl cytosine (5mC)^3^,^4^. In T-even phage, the deoxycytidylate (dCMP) hydroxymethylase (CH) transfers the methylene group from methylene-tetrahydrofolate (CH_2_THF) to the C5 atom of dCMP, and then uses solvent water molecule to hydrate the methylene group to generate hydroxymethyl dCMP (hmdCMP)^5^, a precursor to be incorporated into DNA during replication^6^. Thereafter, its hydroxymethyl group serves as a substrate for glucosylation to form glucosylhydroxymethylated DNA to avoid cleavage by the host restriction systems^7^,^8^.

Some biologically active nucleoside antibiotics, such as bacimethrin^9^, 5-hydroxymethyl blasticidin S^10^ and mildiomycin^11^, also contain 5hmC moieties that are all derived from hmCMP. We previously demonstrated that MilA, a CMP hydroxymethylase in the mildiomycin biosynthetic gene cluster in *Streptomyces rimofaciens* ZJU5119, can convert CMP to hmCMP^12^. HmCMP is then hydrolyzed by MilB to 5-hydroxymethylcytosine (5hmC)^13^, which is finally incorporated into mildiomycin.

MilA and CH are akin to the superfamily of thymidylate synthases (TS), which transfers a methyl group from CH_2_THF to dUMP to form dTMP in the *de novo* thymidylate synthesis pathway and, hence, DNA synthesis^14^. TS is one of the most conserved enzymes in nucleotide metabolism across phyla and therefore is an important drug target. TS from phage T4 (T4 TS) is involved in coordinating DNA synthesis in infected *Escherichia coli* cells^15^. Extensive biochemical and structural studies on TS have provided a wealth of information regarding its catalytic mechanism, specific interactions with dUMP and folate analogs, and stability^14^,^16^,^17^. The structures of TS and CH resemble each other very well, with a root-mean-square-deviation (RMSD) of 1.849 Å for 127 aligned Cα atoms, despite only 24% of sequence identity between them.

Since TS is responsible for the production of dTMP, one of the building blocks for DNA synthesis, it has been extensively studied as a target for cancer chemotherapy^18^. A number of structures of TS in complexes with various fragments of substrates, both in the presence or in the absence of cofactor analogues, are available^19^. These studies revealed that the cofactor triggered closure of the active site, that the pyrimidine ring of the substrate dUMP directed its binding orientation at the active site, that the ribose sugar moiety contributed to the enzyme’s substrate specificity, and that the glycosidic linkage was critical for the precise localization of the substrate^19^. However, structural studies on how TS protein superfamily members differentiate between ribosyl and 2′-deoxyribosyl substrates are relatively limited, probably in part, due to the lack of enzymes in this family biased towards ribosyl substrates. A report in this regard is that the binding affinity of TS for uridine monophosphate (UMP) is 40 times lower than that for dUMP^20^. All other usual members of TS, such as 2′-deoxyuridylate hydroxymethylase (dUH) from phage SPO1^21^, dCMP hydroxymethyalse (CH) from phage T4^22^, dCMP methylase from phage Xp12^23^, are specific for 2′-deoxynucleotides.

Several structural studies on ribose recognition specificity involved in pyrimidine nucleotide metabolism have been reported previously. The human mitochondrial deoxyribonucleotidase mdN prefers the 2′-deoxyribose form of nucleoside monophosphate. In the structure of mdN, a hydrophobic pitch surrounding the 2′ position of the sugar moiety produces an energetically unfavorable environment for the 2′-hydroxyl group of ribonucleoside 5′-monophosphates^24^. Another case of deoxyribose preference is deoxyribonucleoside kinase (dNK) from *Drosophila melanogaster*. In the structure of dNK, the crowded surrounding in the 2′-position of the substrate sugar leads to steric hindrance against the 2′-hydroxyl group and hence makes ribose forms of nucleosides less favorable than deoxyribose forms^25^. A rare case of ribose preference is human uridine-cytidine kinase (UCK). It has high specificity for the 2′-hydroxyl group of pyrimidine ribonucleosides and does not phosphorylate deoxyribose forms^26^,^27^. Comparison of ligand-free and -bound structures of UCK suggested that the ribose needs to be tightly bound to the enzyme in advance and then triggers a considerable conformational change to form the binding site. Poor binding of the deoxyribose sugar moiety cannot produce the induced fit required for the following base recognition and phosphorylation processes^28^. On the other hand, bacterial CMP kinase phosphorylates dCMP nearly as efficiently as CMP. Its structures in complexes with CMP or dCMP showed that Arg181 forms hydrogen bonds with the 3′-hydroxyl of sugar moiety while Asp185 could be hydrogen bonded to both 3′- and 2′-hydroxyl group^29^. There is no hydrophobic pitch or steric hindrance around the 2′-position of the substrate sugar; and unlike UCK, no induced fit is required for base binding. In addition, it was reported that a single Y639F mutation in the T7 RNA polymerase resulted in an ~20 fold loss of its specificity for NTP over dNTP^30^,^31^,^32^; while a single residue Glu710 of *E. coli* DNA polymerase I (Klenow fragment) dictated its specificity for dNTP by sterically blocking the 2′-hydroxyl of an incoming NTP^33^. Besides, the stringency of dNTP over NTP for the MoMLV reverse transcriptase was relaxed from 10,000-fold to merely 30-fold by its F155V mutation^32^, and the dNTP/ddNTP specificities of DNA polymerases of the pol I family could be switched simply by mutating a phenylalanine residue (corresponding to Phe762 for Klenow fragment) to a tyrosine residue^32^.

In this study, we demonstrated that MilA has a substrate preference for CMP (*k*_cat_/*K*_M_ = 39.2 mM^−1^ min^−1^) over dCMP (*k*_cat_/*K*_M_ = 7.84 mM^−1^ min^−1^), and thus offers an opportunity to investigate the mechanism by which conserved TS evolves the preference for ribosyl over 2′-deoxyribosyl groups. The crystal structures of apo MilA, MilA in complexes with CMP, dCMP and hmCMP were determined. Sequence and structure analyses suggested that the selectivity of ribosyl substrates by MilA is attributed to Ala176 and Lys133′ from the other chain of the dimer in the ribose-binding pocket. Mutation of A176S/K133R of MilA resulted in a reversal of substrate preference from CMP to dCMP.

## Results and Discussion

### Substrate preference of MilA for CMP

**Figure 1:**
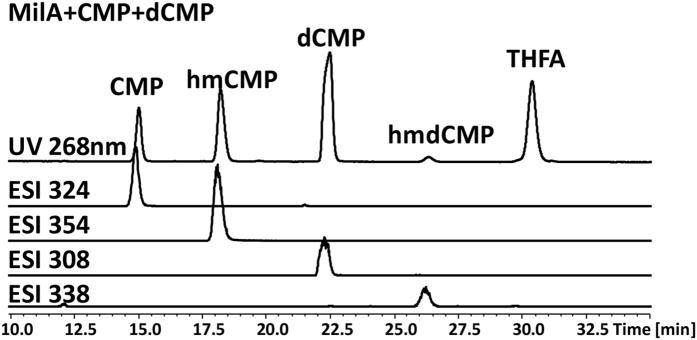
LC-MS analysis of the reaction catalyzed by MilA, with both dCMP and CMP added as substrates to compete with each other. Extracted ion chromatogram at *m/z* 324, 354, 308 and 338 stand for CMP, hmCMP, dCMP and hmdCMP respectively.

We previously reported that MilA could only convert CMP into hmCMP, but could not take dCMP as its substrate^12^. Given only 26% sequence identity with CH, MilA was assayed for hydroxymethylation activity with dCMP as substrate. Unexpectedly, liquid chromatography-mass spectroscopy (LC-MS) detected the ion corresponding to the product hmdCMP ([M + H]^+^ mass = 338, retention time Rt = 16.5 min), however its UV absorption peak was covered by that of the tetrahydrofolate (THFA) (Rt = 16.8 min) (Fig. S1A). To compare substrate preference, equal concentrations of CMP and dCMP were added in the same reaction system with MilA to compete with each other, and hmdCMP and THFA were completely separated using an optimized elution condition in high-performance liquid chromatography (HPLC) analysis. Our results clearly showed that MilA had a strong preference for CMP over dCMP (Fig. 1 and Fig. S1B). The kinetic parameters for MilA were determined with either CMP or dCMP as its substrate (Table 1, Fig. S2). The *K*_M_ for CMP was 0.0719 mM, 3.4-fold lower than that for dCMP (*K*_M_ = 0.245 mM), demonstrating that CMP was a better substrate than dCMP for MilA. The *k*_cat_/*K*_M_ for hmCMP was 39.2 mM^−1^ min^−1^, 5-fold higher than that for hmdCMP (*k*_cat_/*K*_M_ = 7.84 mM^−1^ min^−1^, Table 1). Prompted by this observation, we performed the structural comparison of MilA with CH and other TS members to identify the amino acids of MilA critical for its substrate preference for ribosyl cytidylate.

### Structure of MilA

**Figure 2:**
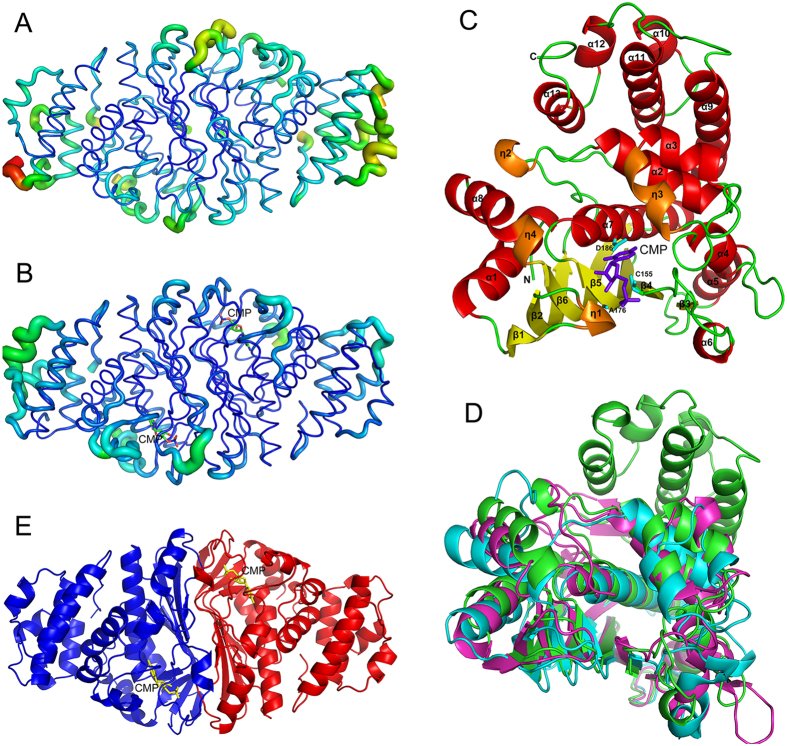
Overall structures of MilA and the MilA‒CMP complex. (**A,B**) The structures of WT MilA (**A**) and the MilA‒CMP complex (**B**) are shown in cartoon representation and colored according to the B-factor. Blue and red represent the lowest and highest B-factor values, respectively. In addition, the thickness of the tube reflects the B-factor value in that the larger the B-factor, the thicker the tube. **(C)** Structure of the MilA‒CMP monomer. α-helices, β-sheets, and 3_10_-helices are colored in yellow, red, and orange, respectively. CMP is depicted in purple. **(D)** Structural comparison of MilA (green), T4 CH (cyan, PDB code 1B5E) and TS (magenta, PDB code 1KZJ). **(E)** Structure of the MilA‒CMP dimer. The structure is viewed perpendicular to the two-fold axis of the dimer. The two protomers are shown in blue and red, respectively. Their bound CMP substrates are represented as yellow sticks.

The structure of C-terminally His-tagged MilA was determined using selenomethionine (SeMet)-substituted MilA-L167M mutant at a 2.20 Å resolution (Table 2). Subsequently, the structures of MilA‒CMP, MilA‒dCMP and MilA‒hmCMP complexes were refined to 1.65 Å, 2.10 Å and 1.80 Å resolution, respectively (Table 2). In the structures of apo MilA and its complexes with various substrates, MilA are all homodimers. The non-crystallographic symmetry (NCS) between the two monomers in the crystallographic asymmetric unit is a twofold rotation with no translation. The N-terminal three residues, C-terminal five residues, residue 232–238 of MilA, as well as the eight residues (LEHHHHHH) introduced by cloning, showed no clear electron density and presumably were disordered in the crystal. The electron density for residues 305–308 was poor in the structure of apo MilA but resolved clearly in the structures of all the MilA‒substrate complexes. There is no obvious difference between the structures of CMP-bound MilA and apo MilA, with the root-mean-square deviation (RMSD) being 0.34 Å for 634 aligned Cα atoms. Interestingly, the average B-factor of a loop region around Arg31 (residues 29–33) is dramatically lowered from 43.2 to 24.8 Å^2^ upon CMP-binding (Fig. 2A & B).

The homodimer of MilA consists of two essentially identical subunits and has approximate dimensions of 108 Å × 108 Å × 112 Å. A MilA monomer consists of a six-stranded β-sheet, surrounded by thirteen α-helices and four 3_10_-helices (Fig. 2C). MilA possesses a common fold shared by TS and CH. Compared with TS and CH, MilA has an extra domain consisting of five α helices (from α9 to α13) in its C-terminal region (Fig. 2C and D). Each active site of the dimer is contributed asymmetrically by residues from both subunits. The substrate CMP is located very close to the dimer interface (Fig. 2E). All six β-strands within each monomer as well as α-helices α1, α5 and α6 are involved in dimerization (Fig. 2C and E), in a manner similar to the dimerization patterns of CH and TS.

### Structural similarity to T4 CH and bacterial TS

**Figure 3:**
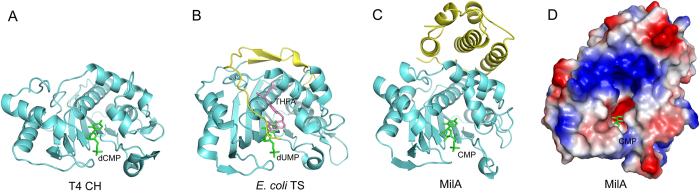
The structure of the C-terminal part of MilA is different from those of T4 CH and *E. coli* TS. **(A–C)** Comparison of the structures of the C-terminal parts of T4 CH (**A**), *E. coli* TS (**B**) and MilA (**C**), which are all colored in yellow. **(D)** The electrostatic surface potential of MilA were generated by pymol, with blue and red representing positively- and negatively-charged surface areas, respectively. The substrate CMP is located in a surface pocket of MilA.

The major parts of MilA, T4 CH and *E. coli* TS subunits resemble each other very well, except for some significant structural difference located at the C-terminal region (Fig. 2D). After getting rid of the the C-terminal region, a superposition of the MilA with *E. coli* TS and T4 CH gives the RMSD of 1.293 Å and 1.209 Å, respectively. *E. coli* TS presents extra 27 C-terminal residues folded as two short β-strands, a 3_10_-helix and a long loop that is absent in CH (Fig. 3A and B). Unlike the structure of CH and *E. coli* TS, the C-terminal region of MilA consists of five helices (α9-α13) linked by loops (Fig. 3C). The last six residues of *E. coli* TS move ~4 Å upon binding folate, and partly cover the active site^34^,^35^. Therefore, the presumable folate-binding site of T4 CH is more open than that of *E. coli* TS^22^. Some parts of this region are believed to provide an interaction surface for dihydrofolate reductase (DHFR)^36^,^37^. However, DHFR is not functionally required to interact with T4 CH and MilA, since tetrahydrofolate is produced in the T4 CH and MilA-catalyzed hydroxymethylation reaction. Different from TS, the extra C-terminal region of MilA is much bigger and positioned away from the active site. C-terminal truncations either from residue 235 or from residue 249 of MilA are both insoluble (data not shown). Presumably, this region could function as a domain to facilitate protein folding.

### CMP binding and ribose specificity

CH, dUH, and TS all prefer the deoxyribose forms of substrates. In contrast, MilA accepts the ribose form more efficiently than the deoxyribose form, which makes it unique. The substrate CMP is bound in a deep active-site pocket of MilA, in a manner similar to the binding of dUMP by T4 CH and TS (Fig. 3). Most of the key amino acids involved in nucleotide recognition between the structures of MilA‒CMP and CH‒dCMP aligned very well, except for several substitutions of amino acids in the binding pocket.

**Figure 4:**
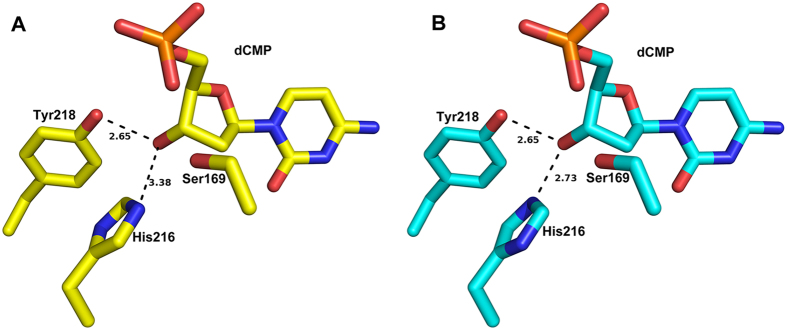
Different rotameric states of His216. **(A)** In the structure of the CH‒dCMP complex determined by Song *et al*., the distance between the ε-nitrogen of His216 and the 3′-oxygen of dCMP is 3.38 Å. **(B)** The alternative rotameric state of His216, with its imidazole ring flipped 180 degrees, is probably more favorable. The distance between the ε-nitrogen of His216 and the 3′-oxygen of dCMP is 2.73 Å.

In the CH structure, His216 and Tyr218 make hydrogen bonds with the 3′-oxygen atom of 2′-deoxyribose sugar^22^. In the crystal structure of CH, the imidazole ring of His216 could be in two different rotameric states. It is the same case for the analogous His216 in MilA. We propose that His216 of MilA and CH both probably adopt the more favorable rotameric state as shown in Fig. 4B, with the distance between the ε-nitrogen of His216 and the 3′-oxygen of dCMP being 2.7 Å rather than 3.4 Å for the other rotameric state.

The catalytic efficiency (as quantified by the *k*_cat_/*K*_M_ value) of MilA for CMP is 5-fold higher than that for dCMP. In contrast, the *k*_cat_/*K*_M_ value of TS for dUMP is about 300-fold higher than that for UMP (Table 1). This observation immediately raises two questions. First, what is the molecular mechanism for that MilA prefers ribose nucleotide substrates whereas TS favors deoxyribose ones? Second, why does TS has a much higher stringency on substrate specificity (with an almost 300-fold difference between the two kinds of substrates) than MilA (with only a mere 5-fold difference)? Both these two interesting questions warrant further investigations for us.

**Figure 5:**
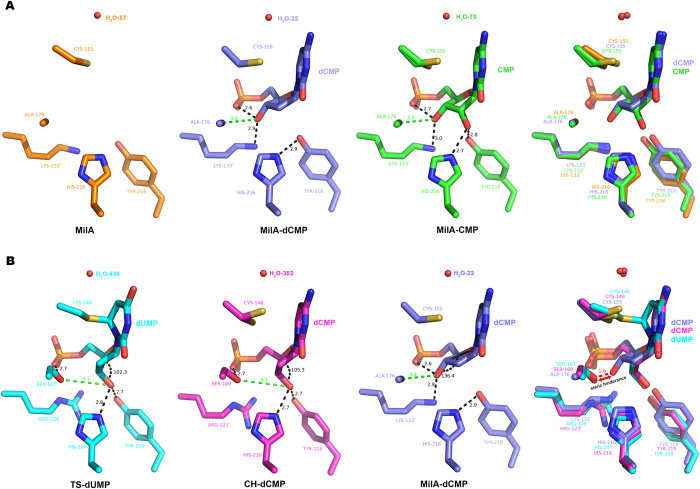
Structural comparison among the substrate-binding sites of MilA alone, the MilA‒CMP complex, the MilA‒dCMP complex, the TS-dUMP complex and the CH‒dCMP complex. (**A**) Structural comparison between the substrate-binding sites of MilA alone, the MilA-dCMP and the MilA‒CMP complex. Panel 1: the substrate-binding site of apo MilA (colored in orange). Panel 2: the substrate-binding site of the MilA‒dCMP complex (colored in slate). Panel 3: the substrate-binding site of the MilA‒CMP complex (colored in tv_green). Panel 4: superposition of the substrate-binding sites of MilA alone, the MilA‒dCMP complex and the MilA-CMP complex. (**B**) Structural comparison between the substrate-binding sites of the TS-dUMP complex, the CH‒dCMP complex and the MilA‒dCMP complex. Panel 1: the substrate-binding site of the the TS-dUMP complex (colored in cyan). Panel 2: the substrate-binding site of the CH‒dCMP complex (colored in light magenta). Panel 3: the substrate-binding site of the MilA‒dCMP complex (colored in slate). Panel 4: superposition of the substrate-binding sites of the TS-dUMP complex, the CH‒dCMP complex and the MilA‒dCMP complex.

Through a comparison of the active site structures of the TS-dUMP, CH‒dCMP, MilA-dCMP and MilA‒CMP complexes, it was found that the 3′-hydroxyl groups of the sugar moiety of substrates adopt two different conformations when complexed with MilA or TS/CH (Figs 5B and S4); the 3′-carbon together with its 3′-OH of deoxyribose motif has a dramatic torsion (with 3′-C set as the vertex, the angle from 6′-O to 3′-O is increased from 102.3°/105.3° to 136.4°) in MilA-dCMP relative to TS-dUMP/CH-dCMP (Fig. 5B, panel 1–3). In TS or CH, both of which prefer deoxyribosyl substrates, the 3′-hydroxyl group of dUMP/dCMP makes hydrogen bonds with TS-His207/CH-His216 and TS-Tyr209/CH-Tyr218 respectively (Fig. 5B, panel 1&2). However, in MilA-dCMP, the 3′-hydroxyl group of dCMP forms one hydrogen bond with the Lys-133′ and another intramolecular hydrogen bond with the phosphate group (Fig. 5B, panel 3). The main reason for this difference is that Ala176 in MilA is replaced by a serine, Ser167/Ser169, in TS/CH. The extra hydroxyl group of TS-Ser167/CH-Ser169 makes the space crowded for the 3′-hydroxyl group of the sugar, and cannot tolerate the sugar moiety of the substrate to adopt the same conformation as that when in complex with MilA. It is not hard to imagine that when UMP or CMP attempts to enter the substrate-binding pocket of TS or CH, the 2′-hydroxyl group of the sugar would occupy the space of the 3′-hydroxyl group, and the 3′-hydroxyl group would have to adopt the same conformation as CMP in MilA, in which case, the additonal hydroxyl group of TS-Ser167 or CH-Ser169 side-chain would give rise to steric hinderance with the 3′-hydroxyl group of the sugar given the close distance (1.9 Å as indicated panel 4 of Fig. 5B). In contrast, Ala176, with its much smaller side-chain methyl group, is the corresponding residue for TS-Ser167/CH-Ser169 in MilA and makes the larger room. Therefore, both CMP and dCMP are able to fit into the substrate-binding pocket of MilA (Fig. 5A). Hence, MilA can not only can utilize CMP, but also can use dCMP as its substrate like CH and TS. This provides an explanation for the second question raised above. An alternative interpretation for this question might be that TS is actually a better enzyme than MilA in terms of catalytic efficiency. According to the kinetics summarized in Table 1, the catalytic efficiency of TS on dUMP is 4 orders of magnitude higher than that of MilA on CMP, probably magnifying the stringency of TS in selection of dUMP over UMP than that of MilA in selection of CMP over dCMP.

**Figure 6:**
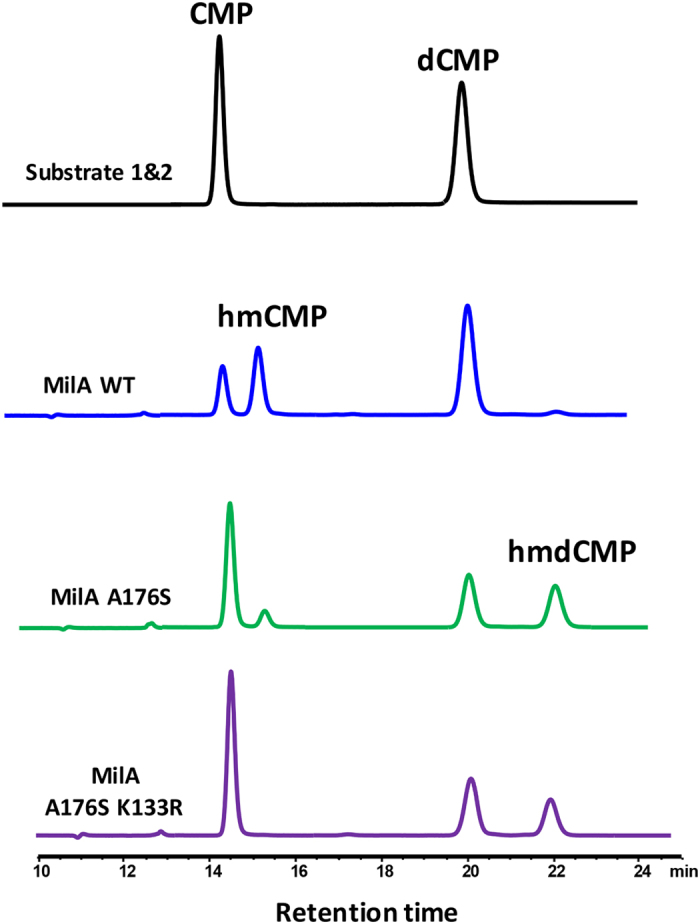
Comparison of the enzymatic activity of MilA-WT, MilA A176S and MilA A176S/K133R towards CMP and dCMP mixture. In the presence of both CMP and dCMP, MilA-WT preferred to hydroxymethylate CMP and just a slight amount of hmdCMP was produced. MilA A176S had dramatically decreased its activity towards CMP, but significantly enhanced its catalytic efficiency toward dCMP. The catalytic efficiency of MilA A176S/K133R toward CMP was completely eliminated, but its efficiency to dCMP is slightly affected.

In accordance with structural analysis, mutation of alanine 176 into serine had dramatically decreased its activity towards CMP, but significantly enhanced its catalytic efficiency toward dCMP (Fig. 6). This further confirmed that Ala176 of MilA is critical for its substrate specificity. The fact that MilA-A176S could still catalyze the hydroxymethylation reaction of CMP implies that its substrate-binding pocket can still accommodate CMP. In the structures of TS-dUMP and CH-dCMP, the guanidino side chain of TS-Arg126′ or CH-Arg123′ could bond to three oxygen atoms of the phosphate group without formation of any bonds to the ribose moiety (Fig. S3A,B). By contrast, its counterpart residue in MilA is lysine 133′, which formed hydrogen bonds with 3′-hydroxyl group of dCMP or CMP in respective protein/substrate complex (Fig. 5A, panel 2&3). It seems that Lys133′ in MilA plays an auxiliary role in the ribose specificity. To address this possibility, Lys133′ was further mutated into arginine on the basis of MilA A176S, the catalytic efficiency toward CMP was completely eliminated in the double mutant MilA A176S/K133R, but its efficiency to dCMP is slightly affected (Fig. 6).

As for the first question, the reason that MilA prefers ribosyl substrates is because in addition to the hydrogen bonds with the 3′-hydroxyl group of CMP, the 2′-hydroxyl group of CMP makes strong hydrogen bonds with Tyr218 and His216 of MilA, with distances of 2.7 Å and 2.8 Å, respectively. These two additional hydrogen bonds make contributions to lower MilA′s *K*_M_ value for CMP compared to that for dCMP. In summary, our structural information strongly implies that the evolution from a serine and an arginine in the active site of TS/CH to an alanine and a lysine in the active site of MilA contributes a lot to the switch of substrate specificity from deoxyribosyl substrate (dUMP/dCMP) to ribosyl substrate (CMP) (Fig. 5).

### Comparison of sequences and identification of critical amino acids

**Figure 7:**
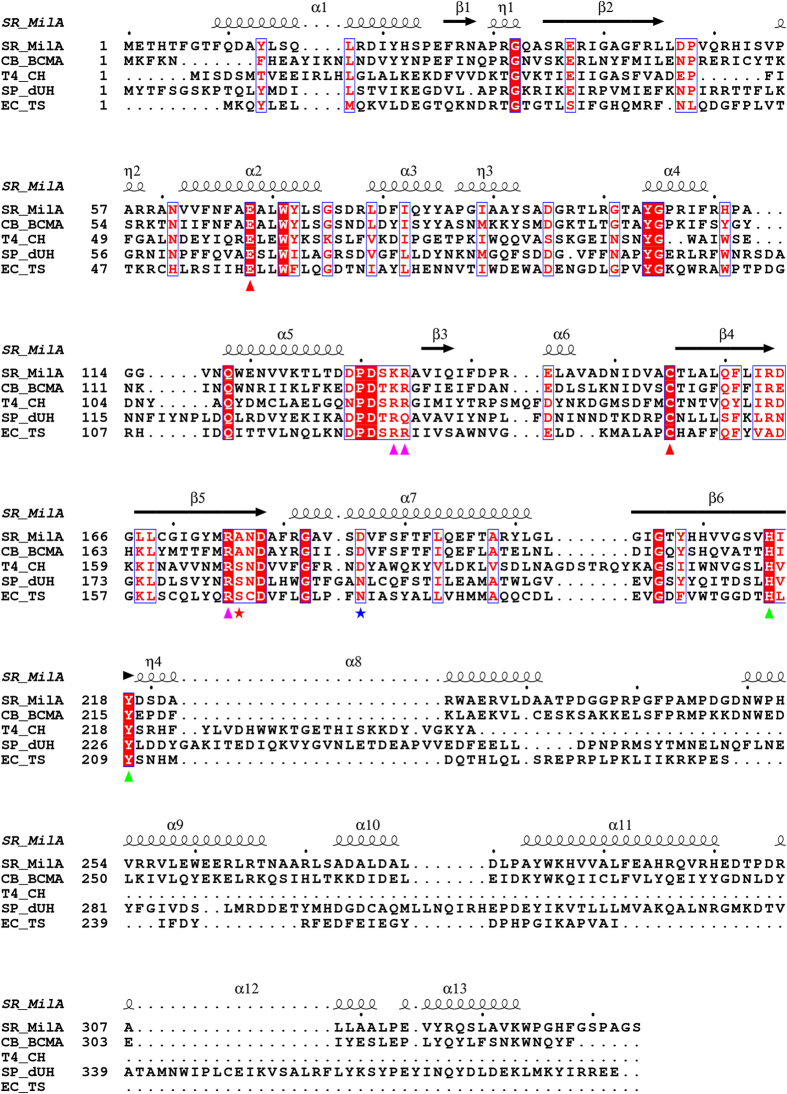
Multiple sequence alignment of MilA and its homologues. MilA from *S. rimofaciens* (SR_MilA), BcmA from *C. botulinum* (CB_BcmA), CH from T4 phage (T4_CH), dUH from phage SPO-1 (SP_dUH) and TS from *E. coli* (EC_TS) were aligned. Conserved residues are highlighted in dark-red background. Residues involved in ribose specificity are indicated with red stars and green triangles. Catalytic residues are represented with red triangles in the bottom. Residues involved in phosphate-binding and base-binding specificities are marked, respectively, with pink triangles and blue star in the bottom. The secondary structure of MilA is shown above the sequences.

CH, dUH and TS all prefer deoxyribose-containing substrates, while MilA and BcmA accept ribose-containing substrates more efficiently than deoxyribose-containing ones. There should be structural differences in the substrate-binding sites of MilA and BcmA from other enzymes. Therefore, we aligned the primary sequences of MilA and BcmA with those of T4 CH, dUH from phage SPO1, and *E. coli* TS using the Cobalt Constraint-based multiple protein alignment tool. The sequence alignment, which is graphically enhanced by Espript 3.0^38^, shows that most of the critical amino acids in the active site are conserved (Fig. 7). For instance, the reactive nucleophile residue Cys155, catalytically important residue Glu68, and ribose-binding residues His216 and Tyr218 of MilA are extremely conserved. These assure similar catalytic mechanisms for MilA, CH, dUH and TS.

On the other hand, three amino acids in MilA, Lys133′, Ala176 and Asp186 are not conserved in all these five proteins. Interestingly, Lys133′ and Ala176 are conserved in MilA and BcmA, which prefer ribosyl-containing substrates; whereas the equivalent residues in enzymes preferring deoxyribosylated substrates are all arginines and serines. The third residue Asp186 is conserved in MilA, BcmA, and T4 CH which utilize cytosine-containing substrates; whereas the equivalent residue in enzymes favoring uracil-containing substrates like TS and dUH are both asparagines. Song *et al*. have proposed that in analogy with *L. casei* TS, Asp179 of T4 CH prefers dCMP to dUMP by achieving a proper orientation of the pyrimidine base through a hydrogen bond network for nucleophilic attack by Cys148 and a better stabilization of the reaction intermediates^22^,^39^, which is consistent with our structure of MilA‒CMP.

## Methods

### Site-directed mutagenesis of MilA

Gene encoding wild type (WT) *Streptomyces rimofaciens* ZJU5119 MilA was cloned into the pET28a (Novagen) vector, with a C-terminal 6 × His tag. All mutant plasmids were produced by the whole-plasmid polymerase chain reaction^40^, and verified by sequencing. The plasmids and the primers used in this study are listed in Supplementary Information, Tables S1 and S2.

### Protein expression and purification

Proteins were overexpressed in the *Escherichia coli* strain BL21(DE3) at 16 °C. 10 ml culture grown overnight from a single colony was inoculated into 1 liter of Luria Broth medium supplied with 50 μg/ml kanamycin and 34 μg/ml chloramphenicol. The culture was incubated at 37 °C to OD_600_ = 0.6~0.8, and induced by 0.2 mM isopropyl β-D-1-thiogalactopyranoside (IPTG) for another 20 hours at 16 °C. The cells were harvested and resuspended in 20 ml binding buffer (20 mM sodium phosphate, pH 7.4, 20 mM imidazole and 500 mM sodium chloride), and lysed by sonication in an ice bath. After centrifugation at 16,000 × g for 30 min at 4 °C, the supernatant was applied to 2 ml Ni-NTA column (Qiagen) pre-equilibrated with the binding buffer. The column was washed by 60 ml binding buffer and 10 ml washing buffer (20 mM sodium phosphate, pH 7.4, 50 mM imidazole and 500 mM sodium chloride). The column was then eluted with 10 ml elution buffer (20 mM sodium phosphate, pH 7.4, 300 mM imidazole and 500 mM sodium chloride). All the eluant was collected and further purified by the Superdex 200 gel filtration chromatography (GE Healthcare) equilibrated with 10 mM Tris-HCl, pH 7.4, 100 mM sodium chloride and 2 mM dithiothreitol. The purified proteins were analyzed by sodium dodecylsulphate-polyacrylamide gel electrophoresis and visualized by Coomassie blue staining, and the protein concentration was determined by using the Bradford Protein Assay Kit (Bio-Rad). The combined peak fractions were concentrated to 10 mg/ml. Selenomethionine (SeMet)-substituted MilA-L167M was expressed using the methionine-autotrophic *E. coli* strain B834 cultured in M9 medium (carbon source: glucose) and purified similarly, except that 20 mM β-mercaptoethanol was added before sonication.

### *In vitro* enzymatic assays of MilA WT and MilA mutants and analytical high-performance liquid chromatography (HPLC)

*In vitro* assays of recombinant MilA were carried out at 37 °C for 1 h in a total volume of 100 μl that contained Tris–HCl buffer (100 mM, pH 7.5), paraformaldehyde (15 mM), 2-mercaptoethanol (50 mM), tetrahydrofolate (2 mM, pH 7.5), CMP and dCMP (1 mM, pH 7.5) and the corresponding His-tagged MilA or its mutants (10 μg). The reactions were quenched by the addition of trichloroacetic acid (4%) on ice, the products were resolved by Agilent TC-C18 column (4.6 mm × 250 mm, 5-Micron) on an Agilent 1200 HPLC system using a mobile phase of a gradient of methanol in water supplied with formic acid (0.1%). The constant flow rate for the LC eluent is 0.3 ml/min. Chromatograms were detected using the absorbance at 275 nm. The percentages of methanol (M) at time t varied according to the following scheme: (t, M), (0, 3), (30, 3), (31, 90), (35, 90), (36, 3), (45, 3). The accurate mass of the reaction products that were previously determined by NMR^12^ were analyzed by QTOF/MS (Agilent G6530A).

### Enzymatic kinetic parameters measurement for MilA

Kinetic parameters were monitored on the basis of production of hmCMP/hmdCMP from CMP or dCMP catalysed by WT MilA. The co-substrate ^5^*N*,^10^*N*-methylenetetrahydrofolate (CH_2_THF) was prepared as reference^41^. As the concentration of CH_2_THF is hard to determine, prior to performing the kinetic assay, CH_2_THF solutions prepared with tetrahydrofolate (THFA) of 2 mM and 5 mM were incubated with 1.6 μM MilA and 2 mM CMP or dCMP, respectively. Compared to the reaction with 2 mM THFA, there is no increase of either product when 5 mM THFA applied, indicating that MilA is saturated with CH_2_THF generated by 2 mM starting THFA. On the other hand, 2 mM CMP or dCMP cannot be completely converted to product in each of reaction. MilA of 1.6 μM was incubated with various concentrations of the substrate in 50-mM Tris–HCl, pH 7.5, for 30 min at 37 °C, and then the reactions (with a total volume of 100 μl) were quenched by the addition of trichloroacetic acid (4%) on ice. After centrifugation at 16,000 × g for 5 min, the samples were analysed by HPLC as described above. The structures of the reaction products were determined by QTOF/MS (Agilent G6530A). Kinetic parameters were calculated by fitting the enzymatic data to the Michaelis–Menten equation by the non-linear regression analysis (Prism5; GraphPad Software Inc.).

### Crystallization

Crystallization trials for full-length MilA were performed at 14 °C using the hanging-drop vapor-diffusion method in 48-well plates. Typically, 1 μl reservoir solution was mixed with 1 μl protein solution and equilibrated against 1 ml reservoir solution. Initial crystallization screening trials were performed using Crystal Screen, Index, PEG/Ion and SaltRx screen kits from Hampton Research. After 2 weeks, small crystals of full-length MilA were obtained from the condition that consists of 30% (w/v) polyethylene glycol 4000, 0.2 M lithium sulfate monohydrate and 0.1 M Tris-HCl, pH 8.5. Longer and thicker crystals were obtained by using 12–20% (w/v) polyethylene glycol 3350. After further optimization, diffracting crystals were obtained from 15% (w/v) polyethylene glycol 3350, 0.08 M lithium sulfate monohydrate and 0.1 M Tris-HCl, pH 8.5, using the hanging-drop vapor-diffusion method in 48-well plates at 14 °C. Given that only two methionine residues are present in MilA, we introduced a L167M mutation into MilA in order to enhance the anomalous diffraction signal. SeMet-MilA-L167M was crystallized at 14 °C in 15% (w/v) polyethylene glycol 3350, 0.08 M lithium sulfate monohydrate and 0.1 M Tris-HCl, pH 8.5. The crystals of MilA‒CMP, MilA‒dCMP and MilA‒hmCMP complexes were obtained by crystallization in the presence of substrates from condition which consists of 0.1 M sodium cacodylate trihydrate, pH 6.5, and 1.4 M sodium acetate trihydrate. The substrate hmCMP was obtained by a one-step conversion of CMP by purified MilA, followed by the purification procedure described as reported^42^. Diffraction datasets of all the crystals were collected at the BL17U1 or BL19U1 beamlines at Shanghai Synchrotron Radiation Facility (SSRF) using an ADSC Quantum 315r CCD area detector and a Pilatus 3–6 M CMOS detector, and processed using HKL2000 and HKL3000^43^,^44^.

### Structure determination

SeMet-MilA L167M crystals belonged to the *P*3_2_21 space group and contained two molecules in the asymmetric unit. Its structure was determined by the single wavelength anomalous diffraction (SAD) method using PHENIX^45^,^46^. Crystals of apo MilA and MilA complexed with its substrates all belonged to the *P*3_2_21 space group, with two molecules in the asymmetric unit. Their structures were determined by the molecular replacement method with Phaser^47^,^48^, using the structure of SeMet-MilA-L167M as the searching model. Model building was performed by Coot^49^ and refinement was performed by REFMAC5^50^ and Phenix^51^. All the data of collection and refinement statistics are shown in Table 2.

## Additional Information

**Accession codes:** The atomic coordinates and structure factors of MilA, MilA‒dCMP, MilA‒CMP and MilA‒hmCMP complexes have been deposited in the Protein Data Bank with accession numbers 5JNH, 5JP9, 5B6D, 5B6E, respectively.

**How to cite this article**: Zhao, G. *et al*. Structural basis of the substrate preference towards CMP for a thymidylate synthase MilA involved in mildiomycin biosynthesis. *Sci. Rep.* **6**, 39675; doi: 10.1038/srep39675 (2016).

**Publisher's note:** Springer Nature remains neutral with regard to jurisdictional claims in published maps and institutional affiliations.

## Supplementary Material

Supplementary Information

## Figures and Tables

**Table 1:** Enzymatic kinetics for MilA, TS and CH.

	Substrate	*K*_M_ (mM)	*k*_cat_ (min^−1^)	*k*_cat_/*K*_M_ (mM^−1^ min^−1^)
MilA	CMP	0.0719 (0.0076)	2.82 (0.028)	39.2 (3.1)
	dCMP	0.245 (0.0589)	1.92 (0.13)	7.84 (1.25)
TS^52^	UMP	1.17 (0.14)	150 (6)	129
	dUMP	0.006 (0.002)	228 (12)	3.80 × 10^4^
CH^39^	dCMP	0.14 (0.05)	892 (88)	6.25 × 10^3^ (327)

**Table 2:** Data collection and refinement statistics.

	SeMet-MilA L167M	MilA	MilA-CMP	MilA-dCMP	MilA-hmCMP
Data collection					
* *Space group	*P*3_2_21	*P*3_2_21	*P*3_2_21	*P*3_2_21	*P*3_2_21
Unit cell parameters					
* a, b, c* (Å)	107.7, 107.7, 112.1	107.8, 107.8, 112.0	109.6, 109.6, 113.4	109.3, 109.3, 113.2	109.3, 109.3, 112.8
* *α, β, γ (°)	90, 90, 120	90, 90, 120	90, 90, 120	90, 90, 120	90, 90, 120
* *Resolution (Å)	50–3.10 (3.21–3.10)	50–2.20 (2.28–2.20)	50–1.65 (1.71–1.65)	50–2.10 (2.18–2.10)	50–1.80 (1.86–1.80)
* R*_merge_ (%)	29.5 (63.6)	15.6 (76.7)	13.2 (88.5)	11.8 (32.9)	11.9 (73.6)
* I*/σ*I*	43.2 (19.4)	23.2 (4.4)	13.6 (1.9)	17.8 (8.1)	19.6 (3.7)
* *Completeness (%)	100 (100)	100 (100)	96.6 (100)	95.8 (100)	100 (100)
* *Redundancy	10.6 (10.9)	21, 8 (19.0)	6.7 (6.9)	11.0 (11.3)	11.2 (11.2)
Refinement					
* *Resolution (Å)		48.02–2.20	48.73–1.65	49.26–2.10	49.24–1.80
* *Unique reflection		38, 499	91, 516	43, 971	68, 810
* R*_work_/*R*_free_ (%)		16.8/20.7	17.2/19.4	15.5/20.6	18.7/20.8
Number of atoms					
* *Protein		5,074	5,067	5,079	5,089
* *Ligand/ion		0	42	40	46
* *Water		336	547	501	201
*B*-factors (Å^2^)					
* *Overall		27.32	24.55	21.6	19.7
* *Protein		26.97	23.4	20.6	19.5
* *Ligand		N/A	18.6	19.2	19.3
* *Water		32.63	35.37	31.93	25.3
* *RMSD bond length (Å)		0.008	0.006	0.007	0.005
* *RMSD bond angles (°)		0.81	0.83	0.86	0.98

Data for each structure were collected or calculated from a single crystal. RMSD, root-mean-square deviations from the ideal geometry. Data for the highest resolution shell are shown in parentheses.
