# Supplementary material for: Structural basis of the substrate preference towards CMP for a thymidylate synthase MilA involved in mildiomycin biosynthesis
Source: Sci Rep. 2016 Dec 21;6:39675. doi: 10.1038/srep39675 (PMC5175136; doi:10.1038/srep39675)
Supplement: Supplementary Information [file srep39675-s1.doc]

Electronic Supplementary Materials for

Structural basis of the substrate preference towards CMP for a thymidylate synthase MilA involved in mildiomycin biosynthesis

Gong Zhao, Cheng Chen, Wei Xiong, Tuling Gao, Zixin Deng, Geng Wu & Xinyi He

State Key Laboratory of Microbial Metabolism and School of Life Sciences and Biotechnology, Shanghai Jiao Tong University, Shanghai 200030 (China). Correspondence and requests for materials should be addressed to G.W. (email: geng.wu@sjtu.edu.cn) or X.H. (email: xyhe@sjtu.edu.cn)

**
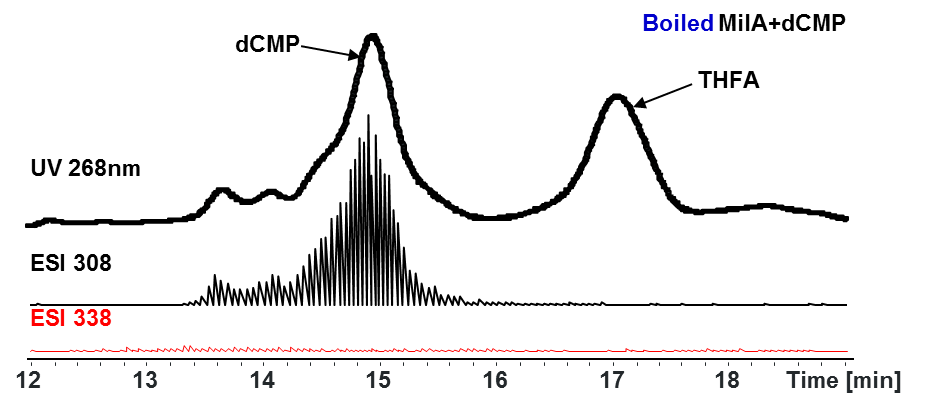

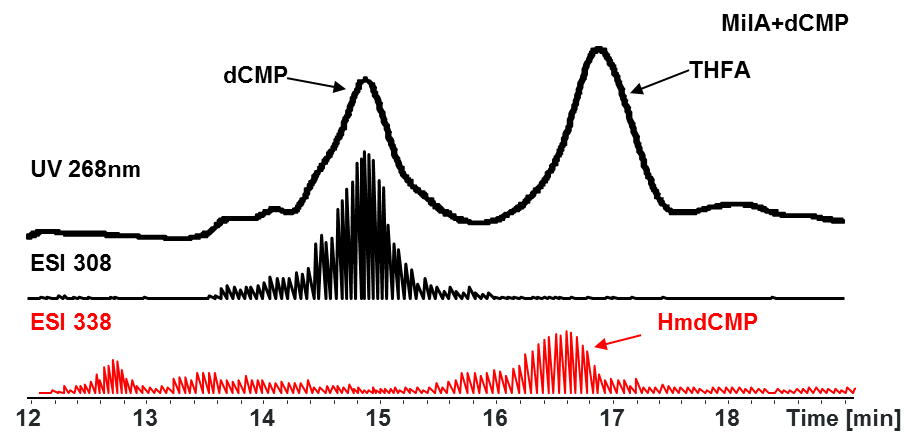
A**


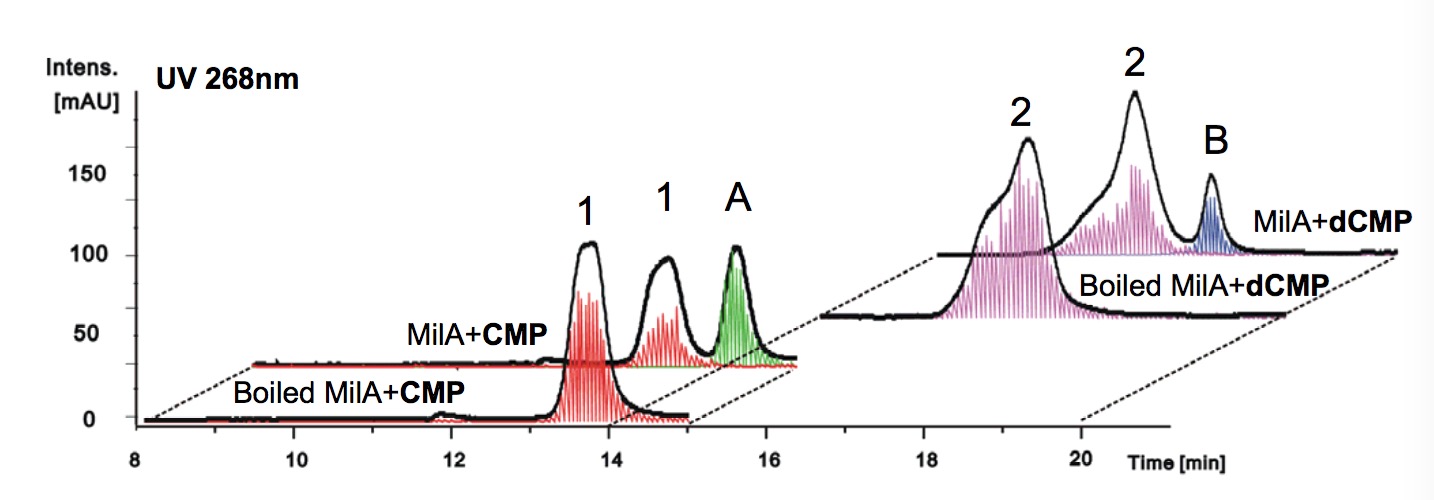
**B**

**Figure S1**. **LC-MS analysis of hmdCMP and hmCMP formation catalyzed by MilA** **and boiled MilA.** **(A)** dCMP can be catalyzed into hmdCMP by MilA, and the UV absorption peak of MilA was covered by that of the tetrahydrofolate (THFA). Extracted ion chromatogram at *m/z* 308 stands for the substrate dCMP, 338 for product hmdCMP. In the right panel, boiled MilA could not react with dCMP. **(B)** Boiled MilA could not convert CMP and dCMP to corresponding products while MilA could make it. In the UV profile, peak 1 and 2 are CMP and dCMP, respectively, peak A and B indicate the absorption of hmCMP and hmdCMP, respectively. lines in red, purple, green and blue correspond to the MS of CMP, dCMP, hmCMP and hmdCMP, respectively.


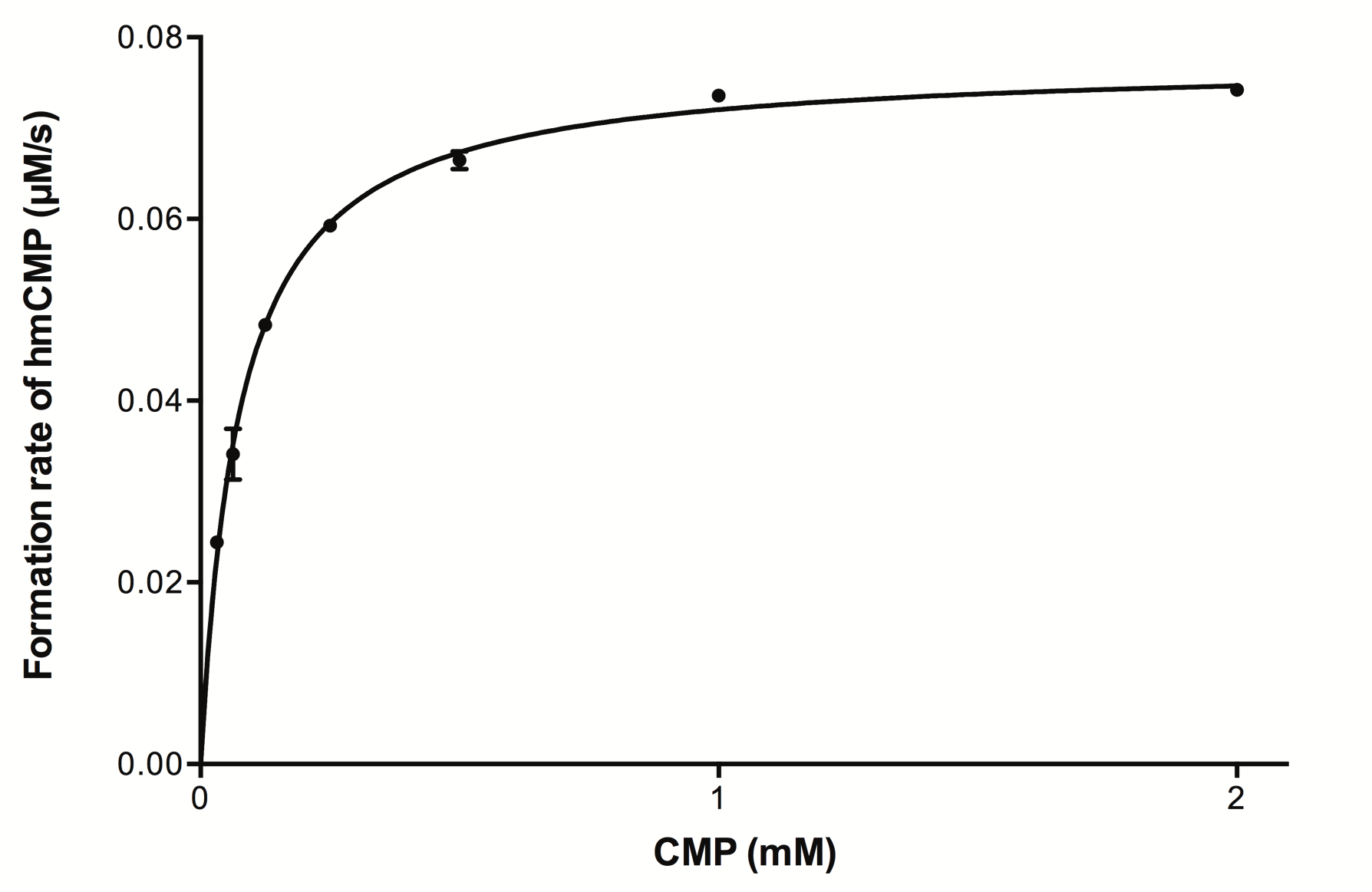

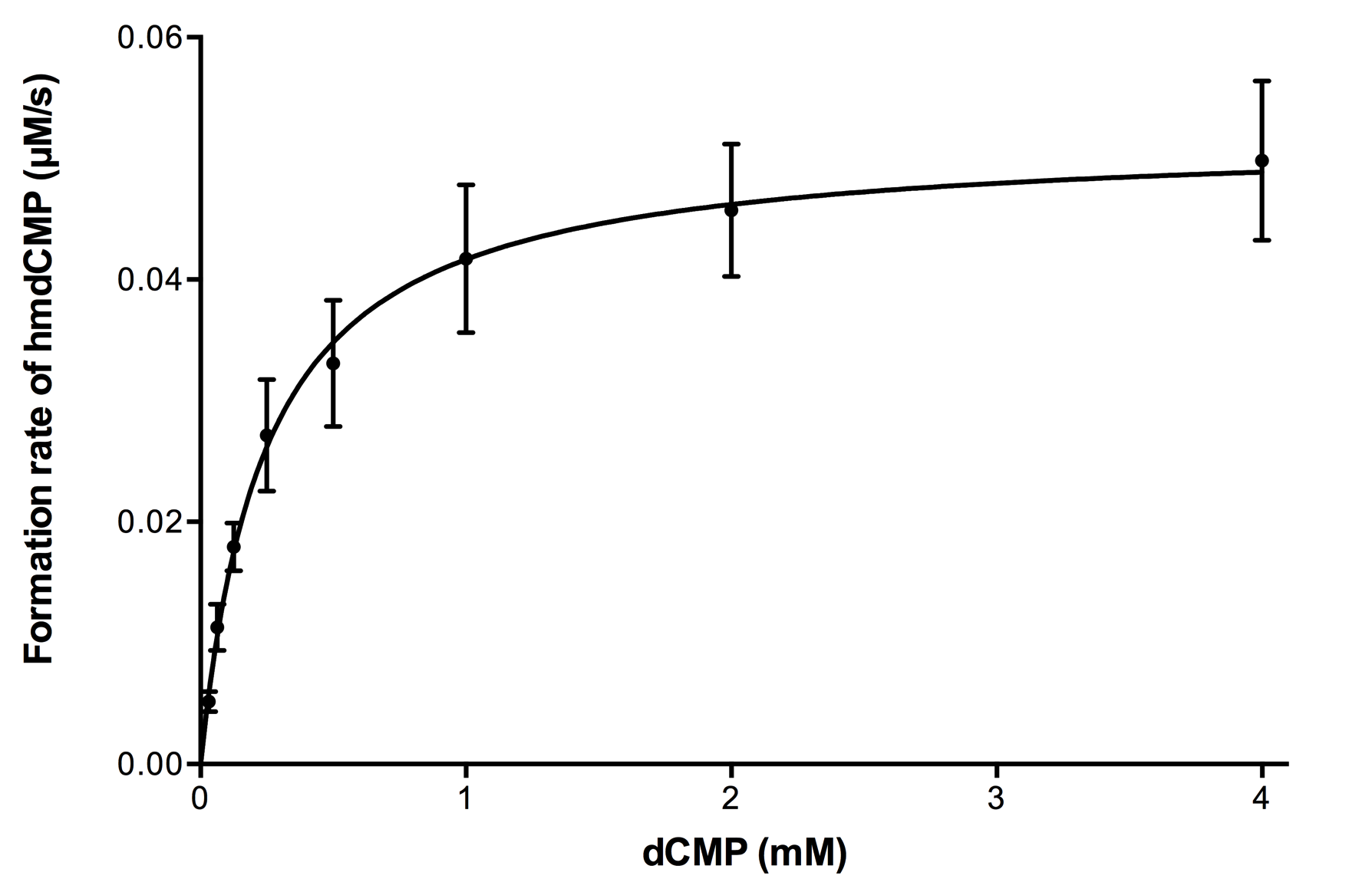


**A**

**B**

**Figure S2. Measurement of the enzymatic kinetic constants of WT MilA, using CMP or dCMP as the substrate. (A)** hmCMP formation catalyzed by WT MilA, with CMP as the substrate. **(B)** hmdCMP formation catalyzed by WT MilA, with dCMP as the substrate.


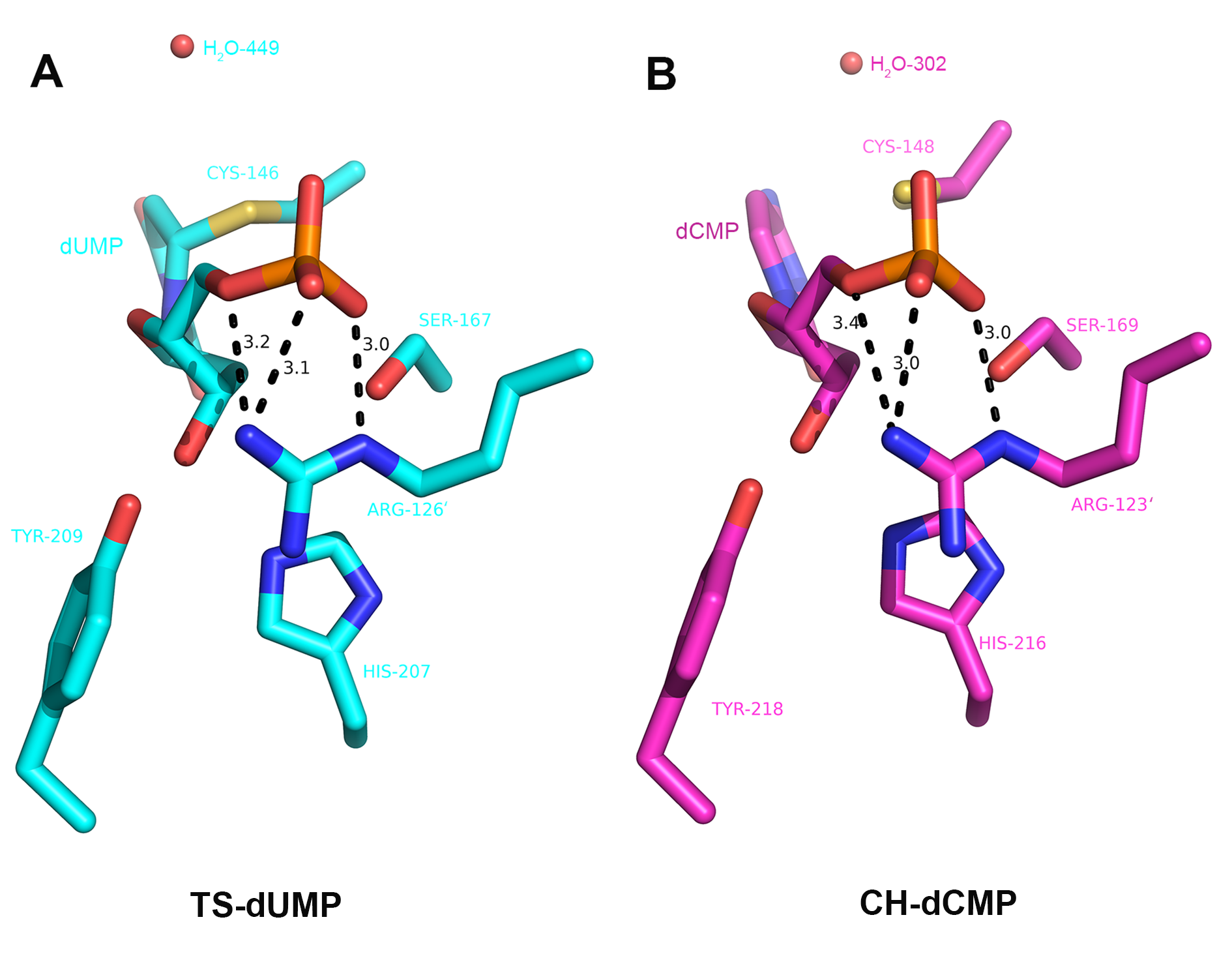


**Figure S3. The conserved arginine recognizes the phosphate group of the substrate in the binding pocket of TS and CH.** In the structures of TS-dUMP (A, colored in cyan, PDB code 1KZJ) and CH-dCMP (B, colored in magenta, PDB code 1B5E), the guanidino side chain of TS-Arg126′ or CH-Arg123′ could bond to three oxygen atoms of the phosphate group without formation of any bonds to the ribose moiety.

**
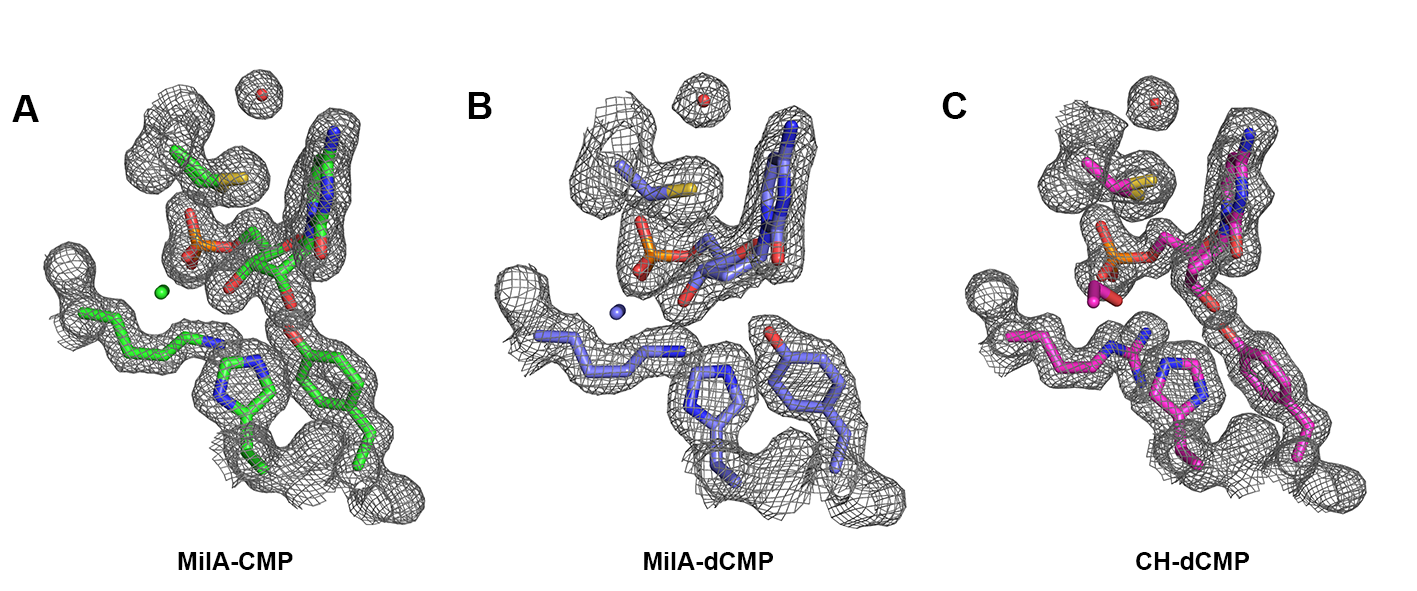
**

**Figure S4. The 2Fo - Fc electron density maps around ribose/deoxyribose binding sites of MilA-CMP (A), MilA-dCMP (B) and CH-dCMP (C, PDB: 1B5E).** The maps are of the crystal structures of the MilA-CMP, MilA-dCMP and CH-dCMP complex, respectively, and are contoured at the 1σ contour level (grey). Models of MilA-CMP, MilA-dCMP and CH-dCMP are colored in tv-green, slate and magenta, respectively. The 3′-hydroxyl groups of the sugar moiety of substrates adopt two different conformations when complexed with MilA or CH.

**Table S1. Plasmids used in this study.**

| **Plasmids** | **Relevant properties** | **Source of reference** |
| --- | --- | --- |
| pJTU2955 | expression vector for MilA, which was cloned into the NcoI and XhoI restriction sites of pET28a(+) | [12] |
| pJTU4397 | MilA L167M expression vector | This study |
| pJTU4398 | MilA A176S expression vector | This study |
| pJTU4399 | MilA 1-235 expression vector | This study |
| pJTU4400 | MilA 1-249 expression vector | This study |
| pJTU4402 | MilA K133R expression vector | This study |
| pJTU4403 | MilA A176S K133R expression vector | This study |

Table S2. Primers used in this study.

|  | **Forward primer** | **Reverse primer** |
| --- | --- | --- |
| MilA_L167M | ctgatccgcgacgggatgctctgcggcatcggc | gccgatgccgcagagcatcccgtcgcggatcag |
| MilA_A176S | tacatgcggtcgaacgacgccttccgggg | ggcgtcgttcgaccgcatgtagccgatgc |
| MilA 1-235 | ccatgcatgccatggaaacccatacgttc | attccgctcgaggtccggcgtcgcggcgtc |
| MilA 1-249 | ccatgcatgccatggaaacccatacgttc | attccgctcgaggtcgccgtccggcatg |
| MilA_K133R | tctgctgtcggggtcgtccgtcagcgtcttg | cgggccgtcatccagatcttcgacccccgg |
